# Case of the Resection of the Superior Maxillary and Malar Bones

**Published:** 1852-08

**Authors:** Daniel Brainard

**Affiliations:** Prof. of Surgery, etc.; Chicago


From the American Journal of the Medical Sciences.

*Case of the Resection of the Superior Maxillary and Malar Bones.* By DANIEL BRAINARD, M. D., Prof. of Surgery, etc.

I was consulted, Oct. 17, 1851, by Caleb Inman, a highly respectable citizen of Wisconsin, residing near Beloit, about a tumor on the left upper jaw. It projected in front below the eye, where there was an ulcerated surface; downward into the mouth, where there was an opening discharging pus and serum; it encroached upon the mastoid, from which there was a discharge of mucus, and projected outwardly beneath the zygomatic arch.

It commenced about eight months previously with a discharge from the nostril. Soon after a tumor appeared beneath the eye, which ulcerated or was opened; it next encroached upon the mouth, and when the teeth fell out, a discharge from the antrum took place. Its growth has been rapid, and attended with much pain.

The patient was sixty years of age, and his health was somewhat impaired.

The treatment had consisted of applications of nit. silver to the ulcerated surface, injections of a solution of it into the antrum, and the iodide of potassium, in solution, given by the mouth.

The operation for its removal was performed Oct. 18, 1851, in presence of Drs. Rutter, McArthur, and several other physicians and students.

The tumor was uncovered by an incision extending from the nose to the zygomatic arch, and another from it to the angle of the mouth, and dissecting up the flaps. The palate portions of the superior maxillary and palate bones were divided by a metacarpal saw, carried into the nostril; the nasal process of the superior maxilla, the connection of the frontal and malar bones, and the zygoma were separated by bone scissors. This loosened the mass, which was so much softened that its removal was completed by the knife.

The hæmorrhage was copious, and required ligatures to beplaced upon numerous enlarged branches of arteries.

It is scarcely necessary to add, that there was great depression resulting from the operation.

The wound having been filled with balls of lint, stitches were applied to the flaps, and the patient put under the influence of opium in full dose.

In aged and debilitated patients always, and in others much depressed by formidable operations, I am in the habit of giving immediately and continuing not only soups and farinacious drinks, but saline food and stimulants. Chicken broth, which contains two per cent. of animal matter to ninety-eight of water, is insufficient; good porter or brandy and meat are necessary.

Under the use of this treatment my patient convalesced without a bad symptom, and in twenty days was able to return home. I have received a line from him dated April 3, 1852, which states that his health is good.

On examining the tumor it seemed to be composed of a congeries of mucous follicles resembling the tonsils, the bony structure being entirely destroyed. It presented no trace of cancerous tissue, and no cancer-cells were detected by the microscope.

CHICAGO, *April* 27, 1852.

